# Chemotherapy in idiopathic pulmonary fibrosis and small-cell lung cancer with poor lung function

**DOI:** 10.1186/s12890-021-01489-4

**Published:** 2021-04-15

**Authors:** Xiyue Zhang, Wei Li, Chunyan Li, Jie Zhang, Zhenzhong Su

**Affiliations:** Department of Respiratory and Critical Care Medicine, The Second Hospital of Jilin University, Changchun, Jilin China

**Keywords:** Small-cell lung cancer, Idiopathic pulmonary fibrosis, Chemotherapy

## Abstract

**Background:**

Idiopathic pulmonary fibrosis (IPF) is a chronic interstitial lung disease with unclear pathogenesis. IPF is considered as a risk factor for lung cancer. Compared to other lung cancers, small-cell lung cancer (SCLC) has a lower incidence, but has a more aggressive course. Patients with IPF and SCLC have a lower survival rate, more difficult treatment, and poorer prognosis.

**Case presentation:**

Case 1 was of a 66-year-old man with IPF for 5 years, who was admitted to our hospital for dyspnea. Case 2 was of a 68-year-old woman, who presented with chest pains, cough, and dyspnea. Both patients had extremely poor lung function. High-resolution computed tomography and pathology revealed that both patients had IPF and SCLC. Chemotherapy comprising nedaplatin (80 mg/m^2^) and etoposide (100 mg for 5 days) was initiated for both patients. Antifibrotic agents were continued during the chemotherapeutic regimen. Both patients showed improvement in their condition after treatment.

**Conclusion:**

The favorable outcomes in these 2 cases suggests that chemotherapy is worth considering in the management of patients having SCLC and IPF with poor lung function.

## Background

Idiopathic pulmonary fibrosis (IPF) is a chronic lung disease that causes irreversible decline in lung function [1]. The changes accompanying IPF severely affect the quality of life of the patients. Furthermore, IPF is associated with a high risk of lung cancer [2]. SCLC is a disease that is generally detected in its later stages, although it is sensitive to chemotherapy. However, in patients with IPF who subsequently develop SCLC, the prognosis is poor. Currently, limited data are available on the treatment of such patients and reports on the successful treatment of such cases are scarce. However, chemotherapy carries the risk of acute exacerbations in IPF patients and contributes to an increased risk of mortality. Therefore, the choice of chemotherapy should be made after careful consideration of the benefits and risks [3]. Herein, we describe two cases of successful treatment in patients with IPF as well as SCLC.

## Case presentation

### Case 1

**Fig. 1:**
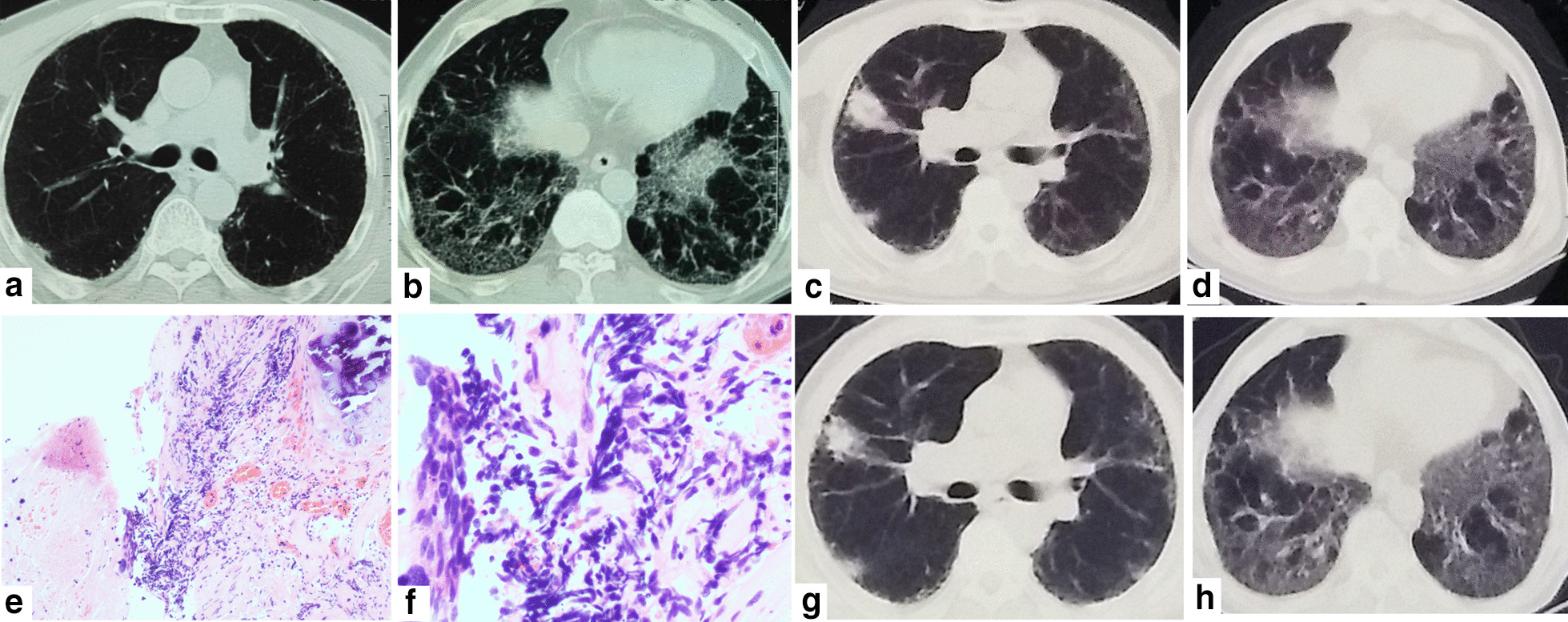
**a**, **b** Chest HRCT scan showed a mass with fibrous stripes and patchy shadows in the lower lobe at the age of 61. **c**, **d** Follow-up Chest HRCT scan at the age of 66 years showed that there were no changes in both the lower lobes, but there was a new space-occupying lesion in the right lung. **e**, **f** Pathological examination (H&E staining, original magnification 100×, 400×) of bronchoscopy sample revealed small-cell lung cancer. G,H, Chest HRCT scan after chemotherapy showed reduction of the shadow in the upper lobe of the right lung

The patient was a 66-year-old man who presented with dyspnea and cough and was referred to the Second Hospital of Jilin University 5 years ago. The patient was a retired worker with a 40 pack-year history of smoking and no history of other diseases. HRCT scan of the chest revealed that the patient had masses in both lungs, with fibrous stripes and diffuse subpleural reticular pattern, which were indicative of IPF (Fig. 1a, b). Lung function tests showed a substantial decrease in the diffusing capacity of the lungs for carbon monoxide (DLCO; < 60%), while blood gas analysis showed reduced levels of partial pressure of oxygen (PaO_2_). Treatment with glucocorticoids and pirfenidone led to improvement of the symptoms. With regular treatment, the patient’s condition continued to remain stable, as confirmed by a follow-up chest HRCT performed 5 years later. However, the follow-up scan revealed a newly developed, irregular mass in the right upper lobe and enlargement of the anterior trachea and posterior vena caval groups of lymph nodes; the lower lobes did not show any changes on either side (Fig. 1c, d). The patient then underwent fiberoptic bronchoscopy for retrieval of a tissue sample. Pathological examination of the sample revealed SCLC in the superior lobe of the right lung (Fig. 1e, f).

A chemotherapeutic regimen comprising nedaplatin (80 mg/m^2^) and etoposide (100 mg for 5 days) was then initiated. However, during the chemotherapy, the administration of antifibrotic agents was continued. After 4 courses of treatment, marked reduction of the tumor was observed. A repeat thoracic CT scan revealed reduction in the size of the shadow in the upper right lung and significant decrease in the size of the metastatic lymph nodes in the anterior tracheal and posterior vena caval groups of lymph nodes; however, there was no visible decrease in the severity of IPF (Fig. 1g, h). Thus, we concluded that the treatment was effective and safe, and we recommend that chemotherapy be continued, depending on whether the patient can tolerate and afford it. We followed up the patient three months later. The patient’s vital signs were stable, and there were no instances of subsequent infections or other complications. Therefore, we concluded that chemotherapy was relatively safe and the patient could benefit from chemotherapy.

### Case 2

**Fig. 2:**
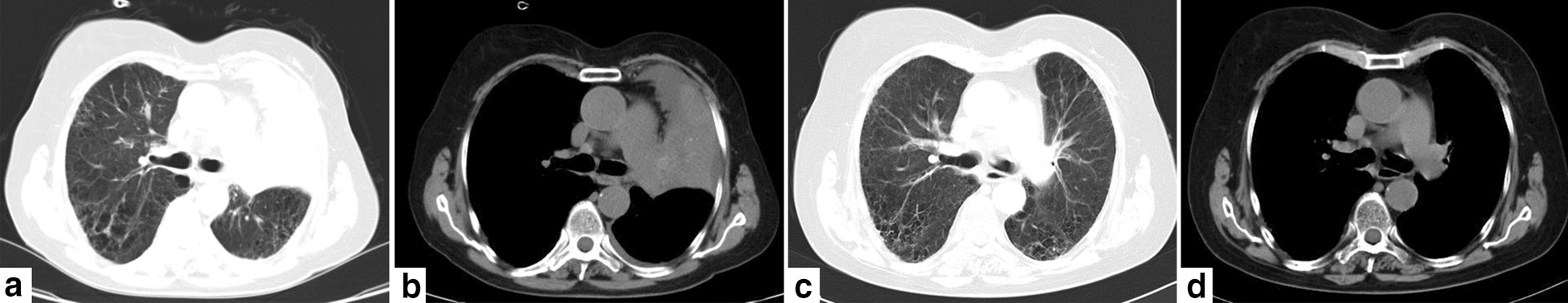
**a**, **b** Chest HRCT scan showed atelectasis of the left lung, pulmonary fibrosis of both lungs and enlarged lymph nodes. **c**, **d** Chest HRCT scan showed absence of atelectasis; reduction of the shadow, and no evidence of enlarged lymph nodes

A 68-year-old woman was admitted to our hospital for the management of chest pains since eight days with no apparent causes. The patient had a 30 pack-year history of smoking and denied having a history of any other diseases. Chest pain was accompanied by cough and dyspnea, which were aggravated after exercise. She had poor lung function (DLCO; < 30%), and blood gas analysis revealed respiratory failure. Chest HRCT scans showed fibrotic changes in the lower lobes of both lungs and high-density shadows in the upper lobe of the left lung with obstructive atelectasis. Further, enlargement of the mediastinal lymph nodes was also noted (Fig. 2a, b). For the identification of the cause of atelectasis, the patient underwent fiberoptic bronchoscopy. The results of pathological examination of the extracted tissue sample were indicative of SCLC. Chemotherapy with intravenous nedaplatin (80 mg/m^2^) and etoposide (100 mg for 5 days) was initiated. During the chemotherapy, treatment with glucocorticoids and *N*-acetylcysteine was continued in order to control the symptoms and prevent the progression of fibrosis. After 6 cycles of chemotherapy, the patient’s symptoms were relieved. Follow-up CT scan revealed a reduction in the extent of atelectasis in the left upper lobe, reduction in the size of the mass shadow, and absence of mediastinal lymph node enlargement (Fig. 2c, d). IPF remained stable. The follow-up was conducted for one year. She was in good condition with no severe dyspnea and no complications such as infection. We conclude that the patient benefited greatly from the chemotherapy.

## Discussion and conclusion

IPF is a severe pulmonary fibrotic disease that entails a high risk of LC; to date, its etiopathogenesis remains elusive [4]. The incidence of LC in IPF patients is relatively high, at 13.54% [5]. Therefore, it is imperative that IPF patients be monitored for the development of LC. Studies have shown some similarities in the pathological features of IPF and LC, including proliferation, immune dysregulation, cell senescence, and resistance to apoptosis [6, 7]. In addition, epithelial changes associated with pulmonary fibrosis may be involved in the development of lung cancer [8]. Smoking is an independent risk factor for lung cancer in IPF patients [9]. Therefore, smokers with IPF, in particular, should be closely monitored for signs of lung cancer. Since treatment measures such as surgery, radiation, and chemotherapy carry the risk of acute exacerbation of IPF and increased mortality, the management of LC in IPF patients is highly challenging. Therefore, the severity of IPF in each patient should be carefully assessed [10]. For patients with IPF, acute exacerbation is a life-threatening event [11]. Non-SCLC has been reported to be commonly associated with IPF, and it is generally treated with nintedanib and pirfenidone [12]. Compared to other cancer types, SCLC has a lower incidence in patients with IPF, but it has a more invasive course and shorter survival [13, 14]. A recent meta-analysis revealed that among IPF patients with lung cancer, the proportion of those with SCLC was 20.48% [5]. In the absence of treatment, patients with SCLC rapidly develop acute exacerbation and have poor survival [15]. However, SCLC is sensitive to chemotherapy. Therefore, chemotherapy may still be a good treatment option for IPF patients with SCLC. Since there have been no adequate clinical trials, the ideal chemotherapy regimen for patients with IPF and SCLC, which can destroy tumor cells without influencing IPF, is yet to be identified.

In Case 1, the patient had a history of IPF since 5 years and insisted on continuing his medication to maintain stability of his condition. In Case 2, IPF and SCLC were detected simultaneously, but the longer history of IPF could not be excluded. The coexistence of IPF and SCLC compromises pulmonary function and may lead to respiratory failure, as seen in Case 2. According to the Eastern Cooperative Oncology Group (ECOG), chemotherapy poses significant risk in patients with IPF. Therefore, before initiation of the treatment, both the patients as well as their family members were explained in detail about the risks and benefits of the treatment, and consent for the treatment was obtained. In both of our cases, the patients benefited from chemotherapy, without the exacerbation of IPF. Adverse events of chemotherapy were defined as low (i.e., grades 1–2), as per the CTCAE v5.0.

Etoposide, in combination with platinum, is a commonly used chemotherapeutic regimen with fairly good treatment outcomes. Nedaplatin is a second-generation cisplatin, with a mechanism of action similar to that of cisplatin, but lower nephrotoxicity and neurotoxicity; additionally, it does not show complete cross-resistance with any other chemotherapeutic agents used in combination with platinum [16]. Etoposide, as a cycle-specific chemotherapeutic drug, is one of the first-line drugs used in the treatment of SCLC. Studies have shown that the objective rate of effectiveness of nedaplatin in combination with etoposide in the treatment of SCLC was 49.3% [17]. Despite these relatively high success rates, the risks associated with chemotherapy cannot be ignored. Etoposide has been shown to induce pulmonary toxicity, and IPF-SCLC is identified as a contraindication for the drug, as per the Japanese package insert [18]. Chemotherapy is likely to induce an acute exacerbation of IPF; therefore, risk–benefit analysis is very important whenever it is considered for patient management. However, currently, there are no other superior treatment options available. The performance status of both our patients was 2; therefore, we planned to initiate chemotherapy with nedaplatin and etoposide, while continuing the antifibrotic agents. Fortunately, the treatment plan resulted in improvement of the patients’ symptoms as well as reduction of the size of tumor shadow and lymph node enlargement evident on HRCT, without any change in the severity of fibrosis. However, despite the treatment success, residual shadows were still present in both patients. This suggested that the course of treatment may not have been adequate. Furthermore, there is also the possibility of other types of lung cancer co-existing, and bronchoscopy may be repeated if necessary.

In conclusion, individualized treatment is necessary for the management of patients with IPF and LC. However, it is necessary to ensure the safety of treatment. Chemotherapy can be considered in patients with poor lung function due to the presence of both IPF and SCLC; however, antifibrotic agents cannot be discontinued during the treatment [19]. Our findings highlight the need for studies on the development of new therapeutic agents that would enable the successful management of both SCLC and IPF.

## Data Availability

The datasets used and/or analyzed during the current study are available from the corresponding author on reasonable request.

## Abbreviations

IPF: Idiopathic pulmonary fibrosis
ILD: Interstitial lung disease
LC: Lung cancer
SCLC: Small cell lung cancer
DLCO: Diffusing capacity of the lungs for carbon monoxide
HRCT: High-resolution computed tomography
CTCAE v5.0: Common Terminology Criteria for Adverse Events v5.0
ECOG: Eastern Cooperative Oncology Group
